# Identification and characterization of extrachromosomal circular DNA in age-related osteoporosis

**DOI:** 10.18632/aging.205388

**Published:** 2023-12-29

**Authors:** Qingrun Zhu, Rudong Chen, Mingjie Kuang, Wen Zhang, Dachuan Wang, Shijie Han

**Affiliations:** 1 Department of Orthopedics, Shandong Provincial Hospital Affiliated to Shandong First Medical University, Jinan 250014, Shandong, China; 2 Department of Orthopedics, The Second Hospital, Cheeloo College of Medicine, Shandong University, Jinan 250033, Shandong, China; 3 Department of Orthopedics, Shandong Provincial Hospital Affiliated to Shandong University, Jinan 250014, Shandong, China

**Keywords:** extrachromosomal circular DNA, osteoporosis, bone metabolism, biomarker, epigenetics

## Abstract

Extrachromosomal circular DNA (eccDNA) was once thought to mainly exist in tumour cells, although it was later shown to be ubiquitous in healthy tissues as well. However, the characteristics and properties of eccDNA in healthy tissue or non-cancer tissue are not well understood. This study first analyses the properties, possible formation mechanisms and potential functions of eccDNA in osteoporotic or normal bone tissue. We used circle-seq to demonstrate the expression spectrum of the eccDNA in the bone tissue. A bioinformatics analysis was performed for the differentially expressed eccDNA, and it enriched the Hippo signalling pathway, PI3K-Akt signalling pathway, Ras signal-ling pathway and other signalling pathways that are closely related to osteoporosis (OP). Then, we used real-time polymerase chain reaction and Sanger sequencing to assess human bone marrow mesenchymal stem cells and obtained the base sequence of the eccDNA cyclization site. Overall, eccDNAs in bone tissue are common and may play a significant role in pathways connected to age-related osteoporosis progression.

## INTRODUCTION

Osteoporosis is a progressive systemic bone disease characterized by low bone mass and bone tissue microstructure deterioration, and age-related fractures are the main clinical consequence of the disease. Millions of people suffer from osteoporosis, and most sufferers are postmenopausal women and elderly individuals. This disease interferes with daily life, reduces patients’ quality of life, and requires a considerable amount of care by medical institutions. Thus, osteoporosis imposes a significant burden on our society [1]. The diagnosis of osteoporosis mainly depends on imaging test. The quantitative assessment of osteoporosis and the estimation of fracture risk rely primarily on bone mineral density (BMD) measurements. BMD can be measured using radiological methods such as dual-energy X-ray absorptiometry (DEXA) and quantitative computed tomography (QCT) [2]. In addition, several other techniques such as high-resolution CT and MR Imaging have been proposed to examine bone structure [3]. Therapies for this disease include osteoporotic drugs (antiresorptive (denosumab and bisphosphonates), anabolic (teriparatide and abaloparatide), antisclerostin (romosozumab), and hormonal (hormone replacement therapy and selective oestrogen receptor modulators)), which are consistently supplemented with adequate nutrition, progressive resistance exercises, and exercise styles related to balance [4, 5]. Currently, the diagnosis of osteoporosis relies on imaging tests, and laboratory methods are lacking. Researchers have been trying to find new ways to test for osteoporosis.

Extrachromosomal circular DNA is the main pattern of extrachromosomal DNA [6]. EccDNA which includes small polydispersed DNA (spcDNA), telomeric circles, microDNA, etc., [7], was first found in mammalian cells by Bassel and Hoota in 1964 [8]. In 1965, Cox et al. reported the discovery of extrachromosomal circular DNA in human tumour specimen cells and called it double minutes (DMs) [9]. Since the 1960s, researchers have found increasing evidence of eccDNA in many species. Yoshiyuki et al. detected single- and double-stranded eccDNA with a length of 200–400 base pairs (bp) from mouse and human cell lines [10]. Researchers generally divide extrachromosomal circular DNA into ecDNA(extrachromosomal DNA) and eccDNA using a length of 100 kb as the boundary [11]. EccDNAs are usually less than 25 kb, have a relatively simple structure and cannot be seen under an optical microscope. EcDNAs often present a size range of the 1–3 Mb or larger, which is sufficient to be visible by optical microscopy. EccDNAs are ubiquitous in tumour tissues, and may contribute to the heterogeneity and evolution of tumours [12]. EccDNA can amplify genes of oncogenes and drug resistance and has been found in tumour tissues as well as blood; thus, it represents a promising biomarker of disease and can be used to judge prognosis [7]. In recent years, studies have shown that eccDNAs are prevalent in healthy human tissue and blood, and may have some link to ageing and other vital activities [6, 13]. Although, few studies have focused on eccDNA and noncancer diseases, scholars have studied eccDNA obtained from peripheral blood mononuclear cells in patients with hepatitis C virus [14]. To date, studies have not focused on eccDNA/ecDNA in osteoporosis, and the characteristics of bone eccDNAs/ecDNAs are unknow. Therefore, the properties, source and function of eccDNA/ecDNA derived from osteoporotic and healthy bone must be further investigated.

## MATERIALS AND METHODS

### Study participants and tissue specimens

This study was approved by the Medical Ethics Committee of Shandong Provincial Hospital Affiliated to Shandong First Medical University, Jinan, China, and it adhered to requirements of the Declaration of Helsinki of the World Medical Association. We recruited six patients with osteoporosis and six with normal bone mineral density in this study (Supplementary Table 1). All patients were healthy men and metabolic and other chronic diseases were excluded. Bone mineral density (BMD) was measured at the lumbar cross section using quantitative computed tomography (QCT) (Supplementary Figure 1). The average age of the osteoporosis group (OP group) was 66 years old and that of the normal group (*N* group) was 68 years old. All patients provided informed written consent. Cancellous bone tissues were obtained from the posterior vertebral column during lumbar spine decompression and fusion surgery due to lumbar disc herniation or lumbar spinal stenosis, and they were snap-frozen in liquid nitrogen and stored at −80°C for preservation.

### Tissue DNA preparation and illumina high-throughput sequencing

High-throughput circular sequencing and subsequent bioinformatics analyses were provided by Genedenovo Biotechnology Co., Ltd., (Guangzhou, China). Briefly, a commercial kit was used to extract genomic DNA according to the manufacturer’s instructions. Qubit (Thermo Fisher, MA, USA) and NanoDrop systems (Thermo Fisher, MA, USA) were used for DNA quality detection. We treated the sample DNA using exonuclease V (New England Biolabs, UK) to degrade the linear genomic DNA and achieve circular DNA enrichment. Based on the restriction enzyme method, MspI (New England Biolabs, UK) was used to digest the resulting cyclic DNA according to the manufacturer’s instructions, and then the next step of library sequencing was performed. Novaseq 6000 was then used to sequence 150 bp (PE150) at both ends of the ecDNA/eccDNA libraries.

### Identification, abundance analysis and genome alignment of eccDNA/ecDNA

For the identification of eccDNA /ecDNA, we used the circular DNA analysis software Circle-Map. Circular DNA with a length of less than 100 kb and between 100 kb and 10Mb was identified as eccDNA and ecDNA respectively. Subsequently, a correlation analysis was carried out independently. According to strict filtration standards, we used FASTP software to obtain high-quality clean reads from raw reads and then analyzed the composition and mass distribution of bases to intuitively display the data quality. High-quality clean reads aligned with the reference genome (Ensembl release 98 GRCh38) using the software BWA (Burrows-Wheeler Aligner). Picard was used to sort the results and mark the duplicated sequences, while bedtools was used to determine the coverage of the genome and statistics on the sequencing depth. Circle-map software was used to detect eccDNA/ecDNA in all samples. Circle-map uses inconsistent read pairs across the cyclic DNA interface to initially locate the cyclic DNA interface location. Subsequently, soft clipped reads (split reads) to define the exact position of the circular DNA interface. Moreover, the software will also take into account the coverage of sequencing readings within the cyclic DNA region and changes in the sequencing depth relative to the surrounding region to determine the reliability of the cyclic DNA.

EccDNA less than 100 kb in length was screened, and split reads greater than or equal to 1 in at least one sample were detected for subsequent analysis. For all detected eccDNAs, we took the sum of each eccDNA split read and discordant read pair as the number of valid interface location tags that supported eccDNA and then calculated the abundance (TPM value) of each eccDNA based on this. For all identified eccDNA, the length distribution of eccDNA from each sample was counted. We used a 50 kb window to calculate the distribution of eccDNA in the whole genome and draw the distribution map of eccDNA fragments on chromosomes. EccDNA was annotated according to eccDNA source regions, and the proportion of eccDNA from various regions was counted. The same analysis method for eccDNA was used to analyse the abundance, origin region, derived genes and expression differences of ecDNA.

### Functional and pathway enrichment analysis of eccDNA-derived genes

Gene Ontology (GO) and Kyoto Encyclopedia of Genes and Genomes (KEGG) analysis were performed for all eccDNAS derived from identified genes. GO functional analysis is performed to provide functional classification annotations of genes and identify significant enrichment among genes. First, we mapped the differentially expressed genes to each term in the GO database (http://www.geneontology.org/) and calculated the number of differentially expressed genes associated with each term to obtain the statistics on the number of differentially expressed genes with certain GO functions. This process was followed by hypergeometric tests, which revealed significant enrichment in differentially expressed genes compared with the whole genome. Pathway significance enrichment analysis took the KEGG pathway as the unit, and hypergeometric tests were performed to identify pathways for significant enrichment of differential genes compared with the whole genome backgrounds. We used significant pathway enrichment to identify the main biochemical metabolic and signal transduction pathways involving different genes.

### Validation of eccDNA through routine polymerase chain reaction (PCR) and sanger sequencing

Many eccDNAs with high expression and expression in multiple samples were selected. We designed the forward primer and reverse primer according to the upstream and downstream base sequences of the junction which can span binding sites, and used these primers for experimental verification. We used type 2 collagenase to extract human bone marrow-derived stem cells (hBMSCs) from human cancellous bone. DNA was extracted from hBMSCs using a Steady Pure Universal Genomic DNA Extraction Kit (Accurate Biotechnology, China), followed by the elimination of linear DNA with T5 Exonuclease (Beyotime Biotechnology, China). ApexHF HS DNA Polymerase FS Master Mix (Akurat Biotechnology, China) was used for PCR. Primer sequences are shown in Supplementary Table 2. The PCR product was then subjected to agarose gel electrophoresis and Sanger sequencing (Tsingke Biotechnology, China). The nucleotide compositions of junctions obtained by Sanger sequencing and high-throughput sequencing were compared.

### Statistical analysis

We used SPSS 26.0 statistical software for statistical description and analyses. The test level was a = 0.05, and *P* < 0.05 was considered statistically significant. The Wilcoxon rank-sum test was used to compare data between two groups. The eccDNA used for the GO and KEGG analysis was screened from within the OP group.

### Data availability statement

The raw sequence data reported in this paper have been deposited in the Genome Sequence Archive in National Genomics Data Center, China National Center for Bioinformation/Beijing Institute of Genomics, Chinese Academy of Sciences (No. HRA002872) that are publicly accessible at https://ngdc.cncb.ac.cn/gsa.

## RESULTS

### Expression profiles of eccDNAs/ecDNAs in normal and osteoporotic bone tissue

**Figure 1:**
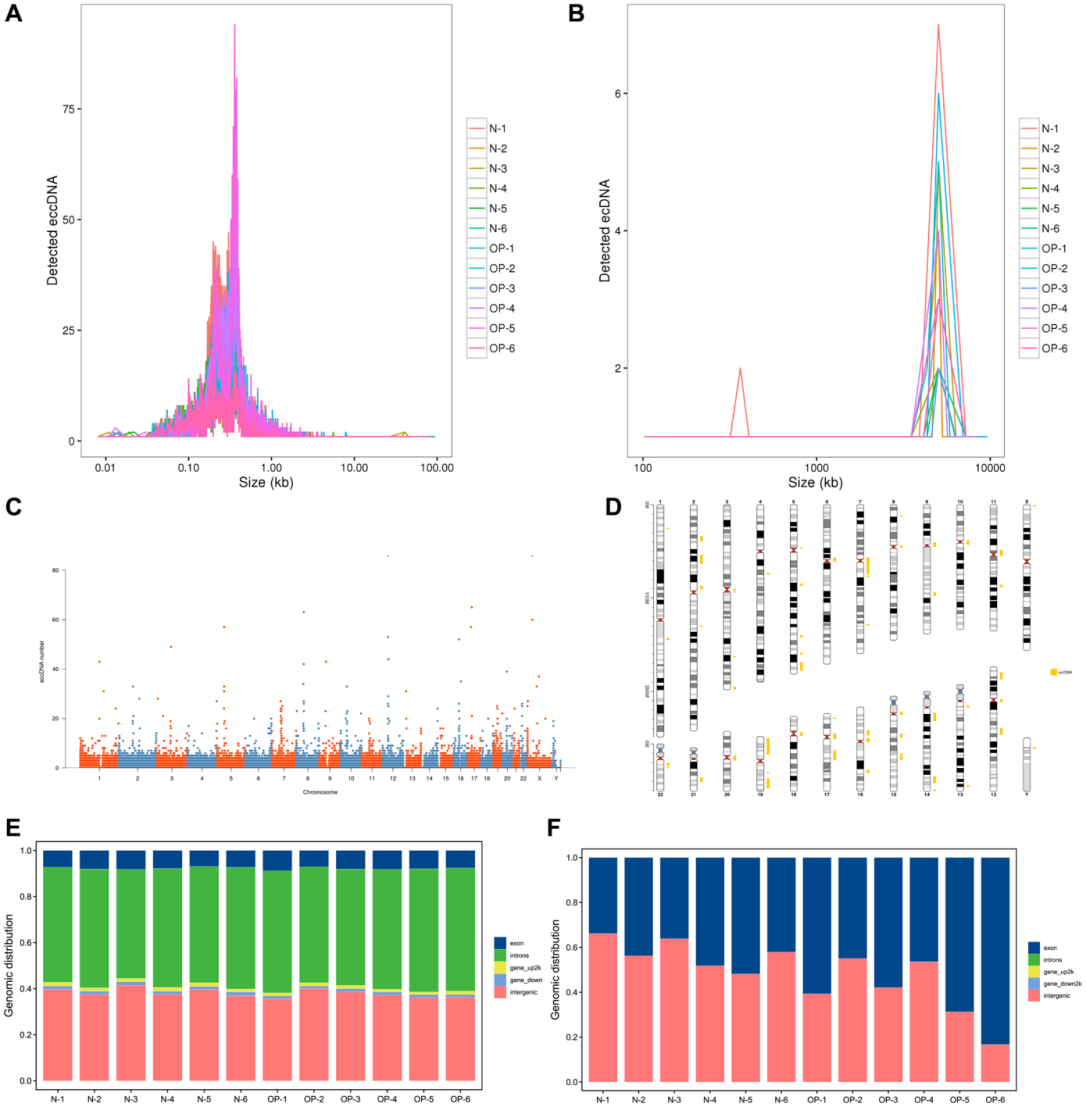
**Length distribution of eccDNAs/ecDNAs and their location on different chromosomes in osteoporotic and normal bone tissue samples.** (**A**, **B**) Length distribution of ecDNAs/ecDNAs in each sample. (**C**, **D**) Distribution of ecDNAs/ecDNAs in the 23 pairs of chromosomes. (**E**, **F**) Distribution of eccDNAs/ecDNAs in different classes of genomic regions.

Large circular DNA usually exceeds 100 kb, while others are mainly below 100 kb. Based on a reference analysis of the relevant literature, eccDNA represented circular DNA from various tissues with a small length and relatively simple structure while ecDNA represented circular DNA with a complex structure and large sequence length [7]. To facilitate subsequent analysis, we used 100 kb as the standard to distinguish eccDNA from ecDNA. The correlation analysis mainly focused on eccDNA. The circular DNA analysis software Circle-Map was used for detection. High-throughput sequencing showed approximately 200 million clean reads per vertebral bone sample. These clean reads were mapped to the human genome (Ensembl release 98 GRCh38) through BWA software, and the comparison results were sorted and repeated sequences were marked using Picard software. Ultimately, 83,545 eccDNAs and 211 ecDNAs were detected in 12 samples (Supplementary Table 3). We found that the eccDNA length varied greatly, but most were ≤5000 bp. Overall, 99.6% of eccDNAs was shorter than 10,000 bp, 99.1% was shorter than 5,000 bp, 98.3% was shorter than 3,000 bp and 92.9% was shorter than 1,000 bp (Figure 1A). These ecDNA molecules showed unimodal size distributions, the vast majority of which were larger than 1,000 kb. (Figure 1B). We separated every chromosome in 50 KB windows, calculated the amount of eccDNA in each window, and revealed the distribution of eccDNA in the whole genome with a Manhattan chart (Figure 1C). The genomic distribution of the eccDNAs shows that they are common in every pair of chromosomes. According to the detected ecDNA component fragments, we plotted the distribution of ecDNA fragments on chromosomes (Figure 1D). We found that circular DNA can come from any chromosome and that chromosome 19 contains more ecDNA than any other chromosome, which may be related to the higher relative density of genes on chromosome 19 compared with other chromosomes [15]. We annotated eccDNA/ecDNA according to the source region of eccDNA/ecDNA and counted the proportion of eccDNA/ecDNA from different genomic elements. The results showed that eccDNA mainly came from introns and intergenic region and the proportion of eccDNA from exons was small. The length of ecDNA indicated that it was derived from both exons and intergenic regions (Figure 1E, 1F).

### Comparing the distribution pattern of eccDNAs/ecDNAs between osteoporotic and normal bone tissue samples

**Figure 2:**
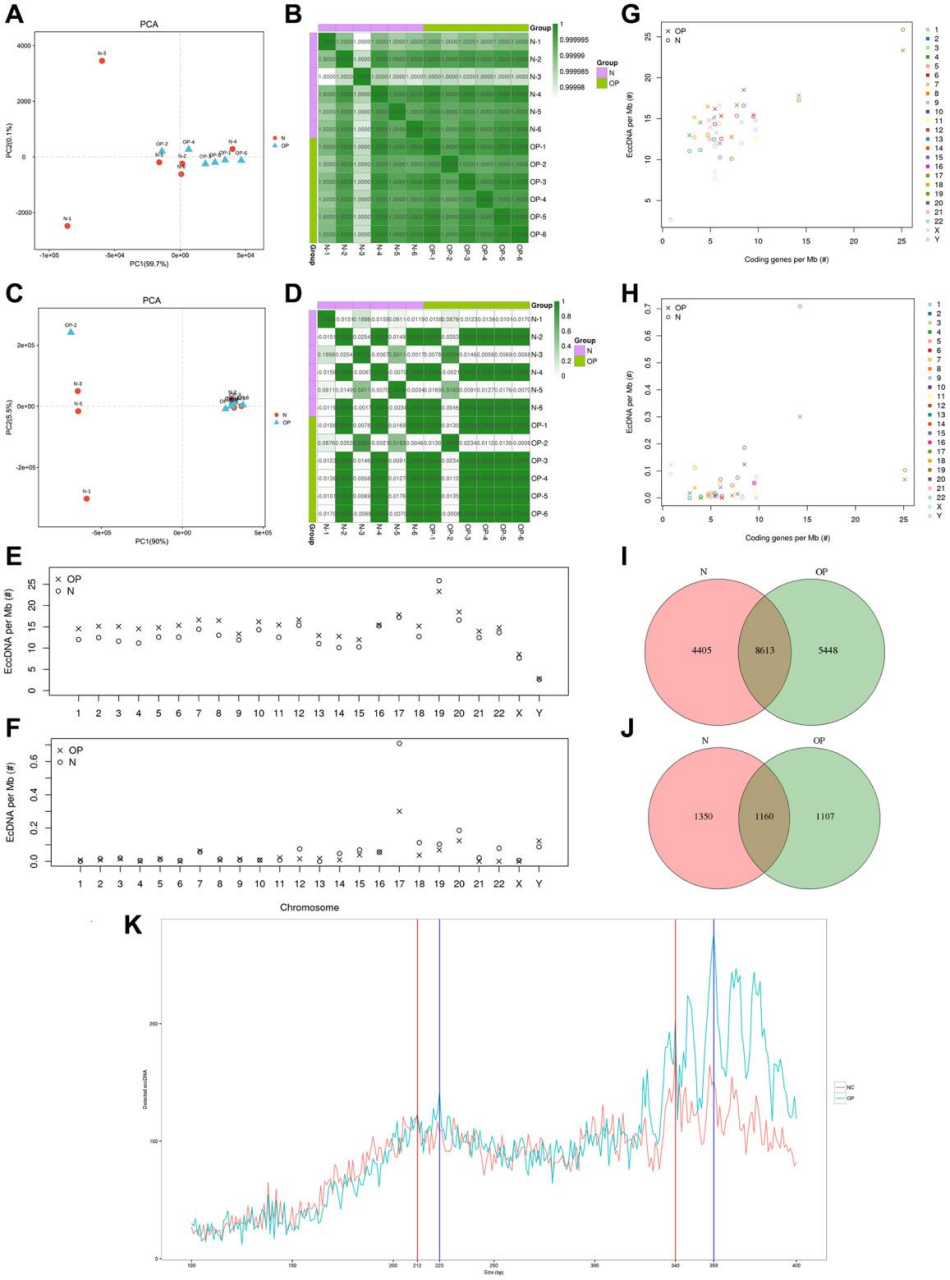
**Sample correlation analysis and comparison of distribution patterns of eccDNAs between osteoporosis and matched normal bone tissue.** (**A**, **B**) PCA and Pearson correlation coefficients based on the profile of eccDNAs of 12 samples. (**C**, **D**) PCA and Pearson correlation coefficients base on the profile of ecDNAs of 12 samples. (**E**–**H**) Ratio of eccDNAs or ecDNAs/Mb and coding genes/Mb in each chromosome. (**I**, **J**) Venn diagram of eccDNAs/ecDNAs detected in the OP and N groups. (**K**) Length distribution of eccDNAs in the OP and *N* groups.

Based on the expression of known genes in each sample, a principal component analysis (PCA) was performed using the R platform (http://www.r-project.org/) to research the distance relationship between samples. The eccDNA/ecDNA expression level in any two samples was used to calculate the Pearson correlation coefficient between each two samples (Figure 2A, 2B). The PCA and Pearson analysis results showed that the osteoporosis group had higher sample similarity than the normal group (Figure 2C, 2D). We analysed the length distribution of eccDNA in the osteoporosis samples and normal samples and identified two main peaks at 223 bp and 359 bp in the OP group and at approximately 212 bp and 340 bp in the normal group (Figure 2K). The amount of eccDNA per Mb in each pair of chromosomes showed that the OP group was generally larger than the normal group except on chromosome 19. The amount of eccDNA per Mb on chromosome 19 was higher than that on other chromosomes, and the amount on the Y chromosome was lower than that on other chromosomes (Figure 2G, 2H). The frequency of ecDNA per Mb of chromosomes in the OP group was not higher than that in the normal group, although the frequency of ecDNA per Mb on chromosome 17 was significantly higher than that on the other chromosomes (Figure 2F). The results are consistent with those previously detected in normal human muscle and blood [7]. Similarly, the coding eccDNAs/Mb and genes/Mb percentages on chromosomes 17 and 19 were significantly higher than those on the other chromosomes (Figure 2E). The same was true for ecDNA (Figure 2F).

For eccDNA/ecDNA originating from regions of coding genes (including exons, introns, gene_UP2K, and gene_down2K), the coding genes are referred to as eccDNA/ecDNA-derived genes. We annotated eccDNA-related coding genes and compared the common and specific eccDNA/ecDNA-derived genes between the normal and OP groups. Previous studies have revealed the highly dynamic nature of these circular DNAs, and a Venn analysis revealed that out of 184,557 eccDNA-derived genes, only 4,405 were detected in normal samples, 5,448 were detected in osteoporosis samples, and 8,613 were detected in both samples (Figure 2I). A total of 3617 ecDNA-derived genes were matched from 211 ecDNA samples (Figure 2J). Most ecDNA contains more than one derived gene, and even one ecDNA (chr19: 45309327-54936985) contains 447 derived genes.

### GO and KEGG pathway analyses of the genes relevant to the differentially expressed eccDNAs

**Figure 3:**
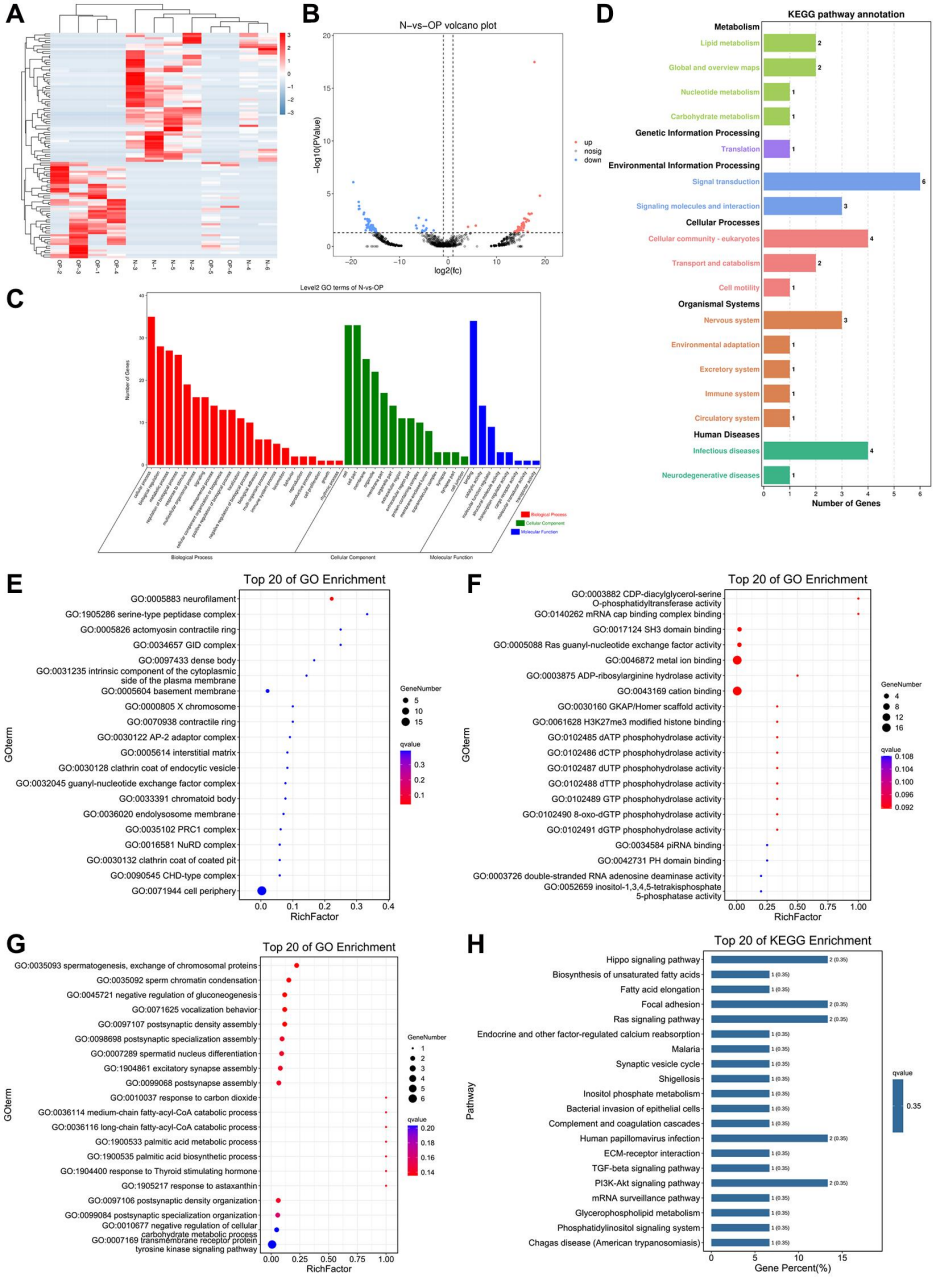
**GO and KEGG pathway analyses of the genes associated with the differentially expressed eccDNAs.** (**A**, **B**) Cluster and volcano plots show eccDNAs differentially expressed in the OP group. (**C**) GO enrichment analysis histogram (the abscissa is the secondary GO term, the ordinate is the number of the genes in this term, and the different tincts represents different types of GO terms). (**D**) KEGG pathway annotation: metabolism, genetic information processing, environmental information processing, cellular processes, organismal systems, human-diseases. (**E**–**G**) Top 20 cell components, top 20 molecular functions and top 20 biological processes. (**H**) KEGG enrichment bar chart (using the first 20 pathways with the smallest *Q*-value to draw this graph, the abscissa is the number of the designated pathway and is a percentage of all differential gene numbers).

Based on the eccDNA/ecDNA differential expression analysis results, eccDNA/ecDNA with a *P*-value < 0.05 and |log2 FC|≥1 was screened as significantly differentiated eccDNA/ecDNA. The results showed that there was only 1 differential eccDNA and 3 differential ecDNA (Supplementary Tables 4, 5), and both were annotated in intergenic regions, which prompted us to change the strategy to analyse the sample information. Subsequently, eccDNAs with 50% similarity within the group and with 50% similarity between the two groups were merged. The Venn analysis was performed based on the combined results. Based on the combined results, eccDNA expressed in at least 3 samples was screened out for the differential analysis, and then the differential eccDNA was screened by DESeq2 with a *P*-value < 0.05 and a twofold difference. A total of 1803 eccDNAs were screened and displayed by cluster (Figure 3A) and volcano plot (Figure 3B).

To detect the potential biological function of eccDNA in the pathogenesis of osteoporosis, go analysis was carried out, including the identification of relevant cellular components, biological processes and molecular functions (Figure 3C). The top 3 terms most enriched in the biological process category were spermatogenesis, exchange of chromosomal proteins, sperm chromatin condensation and negative regulation of gluconeogenesis. The top 3 terms most enriched in cellular components were neurofilament, serine-type peptidase complex, and actomyosin contractile ring. The top 3 terms most enriched in molecular functions were CDP-diacylglycerol-serine O-phosphatidyl transferase activity, mRNA cap binding complex binding and SH3 domain binding (Figure 3E–3G). Moreover, the KEGG significant enrichment analysis was performed to understand the differential genes in the biochemical metabolic pathway and signal transduction pathway (Figure 3D). The KEGG analysis revealed that a total of 39 pathways were enriched, and the results showed that genes connected to the variously expressed eccDNAs were mainly within the Hippo signalling pathway (Figure 3H). The top 20 pathways in the KEGG pathway analysis results were related to osteocytes or osteoporosis.

### Validation of the eccDNAs in the hBMSCs and motif characteristics of the eccDNA junctions

**Figure 4:**
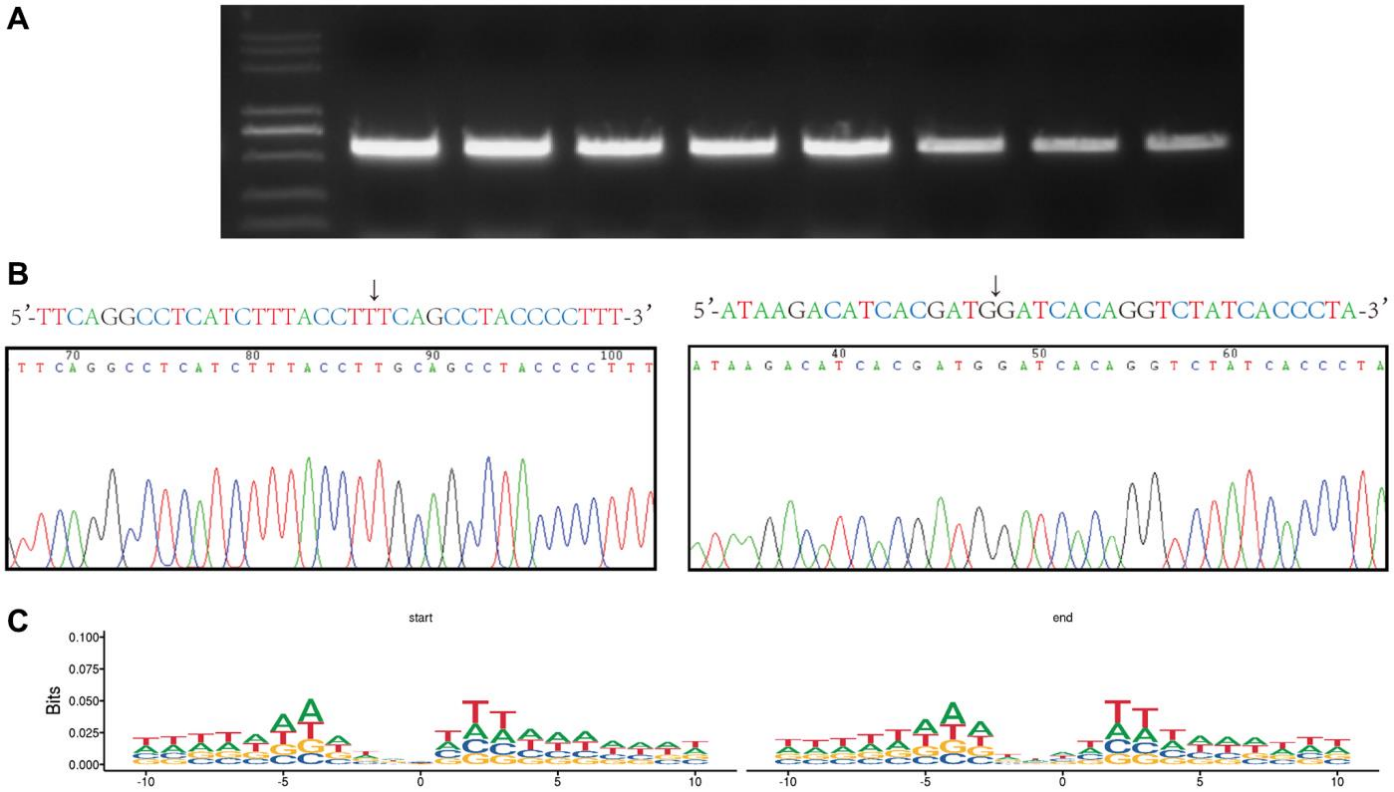
**Verification of the the eccDNA ‘junctional and motif analysis of all eccDNA’ junctional sites.** (**A**) Agarose gel electrophoresis of PCR products from eccDNA. (**B**) Base sequence of the junctional sites we obtained from eccDNA sequencing and Sanger sequencing. The arrow indicates the junction. (**C**) Motif analysis of the junctional sites obtained from the high-throughput sequencing. Consecutive represents ≥three consecutive identical bases at the start and end positions of eccDNA molecules with 4 bp spacers in between.

PCR was performed to process the DNA extracted from hBMSCs, and we found that partially screened eccDNA can be used to reveal the base sequence of the circularization site by performing Sanger sequencing (Figure 4A, 4B). Studies have analysed the base sequences upstream and downstream of the eccDNA junction and concluded that there is a pair of trinucleotide segments on both sides of the start and end position of the eccDNA, with 4-bp “spacers” in the middle [16, 17]. In this study, we used the motif to analyse the nucleotide composition and then each eccDNA starts and end site at 10 bp upstream to 10 bp downstream and then obtained the same trinucleotide segments as previous studies. Interestingly, we obtained longer consecutive sequences of the same nucleotide composition (Figure 4C).

## DISCUSSION

Outer circular DNA, which is generated from chromosomal genes, was once thought to mainly occur in cancer cells, and it is able to promote tumorigenesis [18]. However, Henrik Devitt Møller et al. demonstrated for the first time that eccDNA is ubiquitous in healthy tissue in muscle and blood samples from healthy people. Later, studies proved that eccDNA is ubiquitous in the plasma of pregnant women and oesophageal tissues [16, 17]. In this study, we used high-throughput sequencing to determine a large amount of eccDNA in healthy bone tissue and bone tissue from osteoporosis patients, and our findings supported the above results. We report on the characteristics, distribution, and biological functional correlations of extrachromosomal circular DNA in osteoporotic bone tissue and normal bone tissue, thereby providing a new eccDNA profile for noncancerous human diseases. The identification of eccDNA has provided a new direction for research on the pathogenesis, diagnosis and treatment of osteoporosis. Some studies have proven that eccDNA has specificity for different tissues in the same individual [19]; moreover, the production of eccDNA varies with the stage of development [20]. This finding is similar to the outcome obtained from eccDNA identification in foetus and pregnant mothers [16]. Differences are observed in the length and quantity between the eccDNA identified in our research and those previously identified in the placenta and oesophagus. The eccDNA measuring outcome in our study can be mapped to each human genome region. We compared the distribution of eccDNA in different chromosomes and different regions of gene origin between osteoporotic bone tissue and normal bone tissue, and we did not identify dramatic differences between the groups. However, the eccDNAs/Mb and encoding genes/Mb ratios in each chromosome between osteoporotic bone tissue and normal bone tissue showed certain discrepancies.

The mechanism of eccDNA formation is still unknown. Studies have found that inhibiting protein synthesis with cycloheximide significantly increased the eccDNA content in mouse cells; at the same time, the carcinogen 7,1-dimethylbenzo(a) anthracene and DNA replication inhibitor hydroxyurea, were also increased [21]. Another study found that the level of eccDNA increased after the carcinogen content increased [22]. It is speculated that under physiological conditions, there may be a mechanism to inhibit the production of eccDNA. The findings of these studies are consistent with the tumorigenesis-promoting function [18] of eccDNA. Studies have associated the production of eccDNA with DNA damage repair and found that DNA repair pathways, such as homologous recombination, can promote linear DNA to generate the circles. Researchers have also found that the content of eccDNA decreased significantly after knocking down MSH3 mRNA, which is involved in the DNA mismatch repair pathway in human cancer cell lines [23]. In contrast, another study revealed that the level of eccDNA was significantly increased after knockdown of SGS1 mRNA, which is involved in DNA repair in yeast cells [24]. Several studies have found some regularity in the distribution of bases near the junction of eccDNA [16, 17] and proposed that this regularity may be associated with the microhomology-mediated mechanism of circle formation. We used motif analysis to address the sequence of bases near the eccDNA ligation site and obtained conclusions that were similar to previous studies.

Several studies have demonstrated that amplified oncogenes and drug resistance genes exist in the eccDNA molecules of cancer cells [7, 25, 26]. This trend is associated with intercellular genetic heterogeneity in tumours. EccDNA is homologous to genomic DNA and has the ability to encode complete genes, thus indicating that eccDNA can promote genetic diversity between cells in healthy tissues [2]. In noncancer diseases or normal tissues, the function of eccDNA may be related to ageing [8], immunity [27], etc. Among the top 20 KEGG enriched pathways analysed in our study, the Hippo signalling pathway, Ras signalling pathway, calcium reabsorption regulated by endocrine and other factors pathway, and PI3K-Akt signalling pathway all played a dominant role in the progression of osteoporosis. The Hippo signalling pathway is associated with osteogenic differentiation, osteocyte apoptosis, and excessive osteoclast formation [28, 29]. The Ras signalling pathway is closely related to osteogenesis differentiation and osteoblast proliferation [30] and is also a drug target of bisphosphonates [31]. The PI3K-Akt signalling pathway is a typical pathway for osteoporosis-related research [32], because it is inextricably linked to osteogenesis differentiation, osteoblast proliferation, osteoblast apoptosis, and autophagy and represents the site of action of many drugs. We hypothesized that eccDNA might participate in the progression of osteoporosis through these molecular pathways.

In conclusion, the whole-genome presence of eccDNAs in bone tissue samples was proven by this study. This high-throughput sequencing study is the first to identify and characterize the characteristics of eccDNA associated with osteoporosis. We identified tens of thousands of eccDNAs and analysed the base sequences of their cyclization sites to find characteristic sequences that may contribute to the formation of the cyclization. In hBMSCs outside of the sequencing specimens, we verified the presence of eccDNA identified in sequencing. Bioinformatics analysis found that some molecular pathways associated with eccDNA that differed in the osteoporosis group are closely related to the progression of osteoporosis. Currently, we still need larger sample sizes to further validate these findings. We found specific features of eccDNA in osteoporosis, which contribute to the search for biomarkers for osteoporosis and therapeutic targets. The unique circular structure of eccDNA makes it more stable than other extrachromosomal nucleic acid molecules and can even be helpful for the treatment and prognosis of osteoporosis. Further research on eccDNA will be carried to ascertain the exact mechanisms leading to the differential expression of eccDNA in osteoporosis. Overall, research on eccDNA has revealed the plasticity of genomic DNA and the dynamics of gene amplification, thus increasing our understanding of these important functions. However, additional research on these understudied molecules and their associated mechanisms is still required research on these understudied molecules and their associated mechanisms is still required.

## Supplementary Materials

Supplementary Figure 1

Supplementary Tables
