# Supplementary figures and images for: Identification and characterization of extrachromosomal circular DNA in age-related osteoporosis
Source: Aging (Albany NY). 2023 Dec 29;15(24):15489–503. doi: 10.18632/aging.205388 (PMC10781488; doi:10.18632/aging.205388)

## SUPPLEMENTARY FIGURE

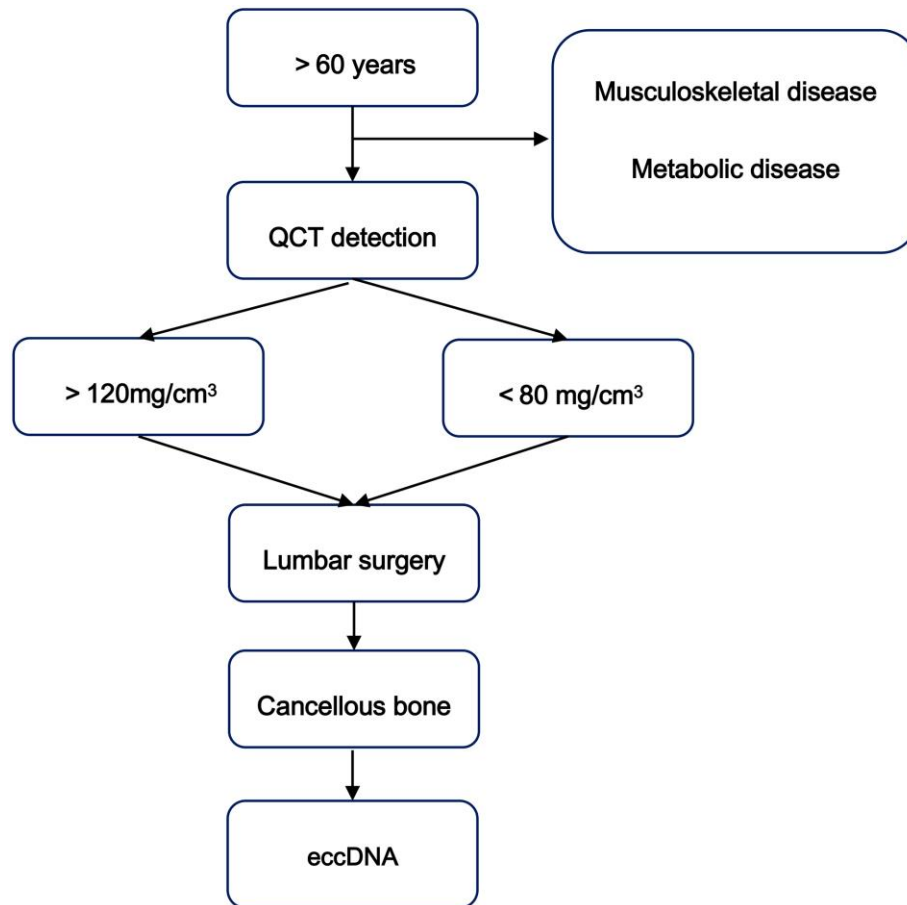

Supplementary Figure 1. The workflow for identification of the 12 patients.

Supplement: Supplementary Figure 1 [file aging-15-205388-s001.pdf]
