# Supplementary material for: Identification and characterization of extrachromosomal circular DNA in age-related osteoporosis
Source: Aging (Albany NY). 2023 Dec 29;15(24):15489–503. doi: 10.18632/aging.205388 (PMC10781488; doi:10.18632/aging.205388)
Supplement: Supplementary Tables [file aging-15-205388-s002.pdf]

SUPPLEMENTARY TABLES

Supplementary Table 1. Patient's information.

Traits	OP						*N*					
	1	2	3	4	5	6	1	2	3	4	5	6
Age (years)	52	74	73	72	75	67	52	74	69	69	69	67
Gender	male	male	male	male	male	male	male	male	male	male	male	male
BMD	75.2	70.9	76.6	72	76.3	46.3	147.9	120.4	120.1	120.1	120	143.3
T value	−3.67	−3.82	−2.98	−3.15	−3	−4.03	−1.07	−1.48	−1.49	−1.48	−1.47	−0.69

Supplementary Table 2. Primer sequences.

ID	Forward primer	Reverse primer
**eccDNA00290067**	CCTTTGAACTTGCCTGTGCC	AGCCATTTGAACCCCGTTCT
**eccDNA00087397**	ATTGAGGGACGGAAAGGCAG	ATTGAGGGACGGAAAGGCAG
**eccDNA00199129**	TTCTCCTTCTGTTCCGGCAG	GCAGGAAGATGTGGAGACAG
**eccDNA00308443**	CCTCCTCCTGCCTACTCCTT	CCACTGAGGGCTTGATCAAT

Supplementary Table 3. The number of eccDNAs and ecDNAs in each sample.

Reads or count	OP-1	OP-2	OP-3	OP-4	OP-5	OP-6	N-1	N-2	N-3	N-4	N-5	N-6
Raw reads	222,931,690	181,936,082	178,309,534	186,710,110	249,405,122	234,999,100	199,049,546	180,378,962	178,980,704	179,698,948	182,324,782	261,230,834
Clean reads	172,443,856	104,399,893	120,313,907	133,659,770	198,986,324	205,949,091	98,115,394	119,929,334	99,552,556	140,441,823	120,060,065	206,130,314
eccDNA count	6,712	8,839	7,648	9,105	10,125	3,310	10,884	6,792	5,461	4,780	6,115	5,291
ecDNA count	28	40	19	28	16	12	68	32	47	27	27	19
eccDNA derived genes	4,210	4,800	4,440	5,095	5,511	2,182	5,855	4,132	3,188	3,096	3,619	3,234
ecDNA derived genes	695	1001	570	359	348	377	976	706	682	326	1,279	511

Supplementary Table 4. The amount of significantly differentiated eccDNA/ecDNA.

eccDNA/ecDNA	N-1 count	N-2 count	N-3 count	N-4 count	N-5 count	N-6 count	OP-1 count	OP-2 count	OP-3 count	OP-4 count	OP-5 count	OP-6 count
eccDNA (chrY:10960687-11028340)	8	4	12	0	7	0	0	0	0	0	0	0
eccDNA (chr15:18360676-19640574)	30	0	60	18	0	0	0	0	0	2	0	0
eccDNA (chr15:18405162-19640574)	5	0	33	0	3	12	0	0	0	0	0	0
eccDNA (chr15:18910372-19640601)	53	0	22	0	0	3	0	0	0	0	0	0

Supplementary Table 5. The clean reads of significantly differentiated eccDNA/ecDNA.

eccDNA/ ecDNA	N-1 TPM	N-2 TPM	N-3 TPM	N-4 TPM	N-5 TPM	N-6 TPM	OP-1 TPM	OP-2 TPM	OP-3 TPM	OP-4 TPM	OP-5 TPM	OP-6 TPM
eccDNA (chrY: 10960687-11028340)	58.7651963125	24.9081511925	143.046168151	0	55.2037412364	0	0	0	0	0	0	0
ecDNA (chr 15: 18360676-19640574)	51369.8630137	0	111731.843575	3182.46110325	0	0	0	0	0	663.349917081	0	0
ecDNA (chr 15: 18405162-19640574)	8561.64383562	0	61452.5139665	0	10638.2978723	2361.739815	0	0	0	0	0	0
ecDNA (chr 15: 18910372-19640601	90753.4346575	0	40968.3426443	0	0	590.434953749	0	0	0	0	0	0
